# Newly diagnosed, treatment-naive patients with rheumatoid arthritis have early abnormalities of vascular and myocardial function

**DOI:** 10.1186/1532-429X-17-S1-P285

**Published:** 2015-02-03

**Authors:** Bara Erhayiem, Adam K McDiarmid, Peter P Swoboda, Ananth Kidambi, David P Ripley, Tarique A Musa, Laura E Dobson, Pankaj Garg, Jacqueline Andrews, John P Greenwood, Maya H Buch, Sven Plein

**Affiliations:** Leeds Institute of Rheumatic & Musculoskeletal Medicine, University of Leeds, Leeds, UK; Multidisciplinary Cardiovascular Research Centre & Leeds Institute for Cardiovascular and Metabolic Medicine, University of Leeds, Leeds, UK

## Background

Rheumatoid arthritis (RA) is a common autoimmune disease. Mortality is increased compared to the general population largely due to increased cardiovascular disease (CVD). Immune dysregulation and systemic inflammation are thought to be associated with this increased risk. CMR can provide an assessment of CVD, including myocardial and vascular morphology and function, but it is unknown which CMR measurements are most sensitive to detect early CVD.

## Objectives

To determine CV manifestations of RA using CMR in newly diagnosed, treatment-naive patients compared with matched controls.

## Methods

Fifty-two RA patients fulfilling ACR/EULAR classification RA criteria, without CVD history, underwent CMR at 3.0T (Philips Achieva TX). They had symptoms for less than 1 year, had not yet received therapy with any disease modifying anti-rheumatic drugs, disease activity score (DAS28) ≥ 3.2 and at least one RA poor prognostic factor. 30 healthy controls were matched by age, sex and blood pressure. Standard balanced steady state free precession (SSFP) cine images were acquired and LV dimensions calculated. Strain analysis was performed by tissue-tagging using a spatial modulation of magnetization pulse sequence. For aortic distensibility (AD), multi-phase SSFP cine images (50 phases) were acquired in a plane transverse to the ascending aorta at the level of the pulmonary artery bifurcation. Aortic contours were drawn by manual planimetry of the endovascular-blood pool interface at the times of minimal and maximal distension. Native and 15 minute post-contrast T1 maps were generated from mid-LV short axis using a modified 3,3,5 Look-Locker inversion sequence to calculate extra-cellular volume fraction (ECV).

## Results

Participant characteristics are presented in Table 1.

**Table 1:** Participant characteristics according to Rheumatoid Arthritis status.

Characteristic	RA n = 52	Control n = 30	p-value
Age, years	49.3±14.1	46.7±11.4	0.39
Female gender; n (%)	34 (65)	15 (50)	0.18
Body surface area, m^2^	1.87±0.22	1.9±0.21	0.43
Systolic blood pressure, mmHg	124±17	125±16	0.80
Pulse pressure, mmHg	54±13	53±13	0.68
ESR, mm/h, median (IQR)	31.5 (34)	-	-
CRP, mg/L, median (IQR)	6.4 (34)	-	-
ACPA, n (%)	44 (85)	-	-
RF, n (%)	41 (79)	-	-
DAS28	5.8±1.7	-	-

Data presented as mean±SD unless otherwise stated. ACPA= anti-citrullinated peptide antibody, CRP= C-reactive protein, DAS= Disease activity score, ESR= Erythrocyte sedimentation rate, RA= Rheumatoid arthritis, RF= Rheumatoid factor.

Aortic distensibility was reduced in RA patients compared to controls (mean±SD, 3.23±1.6 10^-3^mmHg^-1^ versus 4.99±1.8 10^-3^mmHg^-1^, p=0.001). Other measures of arterial stiffness including aortic stiffness index, compliance and strain showed differences with similar significance levels. Left ventricular remodelling was not different in the RA group compared with controls, but S' was lower in RA (mean±SD, 0.99±0.22Seconds^-1^ versus 1.13±0.16Seconds^-1^, p=0.007) which may signify sub-clinical reduction in myocardial performance. Evidence for overt inflammation/fibrosis was seen in 4 patients with areas of focal non-ischaemic patterns of LGE, but native T1 and ECV was not different between the groups. See Table 2.

**Table 2:** CMR findings between RA patients and controls.

Measurement	RA n = 52	Control n = 30	p-value
Aortic distensibility, 10^-3^mmHg^-1^	3.23± 1.6	4.99±1.8	**0.001**
Aortic compliance	11.5±4.7	16.7±5.2	**<0.001**
Aortic strain	-0.16± -0.07	-0.25± -0.06	**<0.001**
Aortic stiffness index, ß	4.53±2.69	2.33±0.71	**0.001**
LVEDV, ml	153±36	167±31	0.082
LVEF, %	60±6	61±4	0.504
LV Mass, g	83±23	90±4	0.216
LV Mass/LVEDV, g/ml	0.55±0.11	0.54±0.11	0.696
S', Seconds^-1^	0.99±0.22	1.13±0.16	**0.007**
Peak twist, degrees	15.6±4.2	14.16±4.7	0.118
Torsion, degrees	13.9±3.6	13.1±4.1	0.211
LGE, n (%)	4 (8)	0 (0)	**0.044**
Native T1, ms	1179±45	1160±50	0.081
ECV, %	27.0±3.9	25.8±2.6	0.213

Data presented as mean±SD unless otherwise stated. ECV=extra-cellular volume, EDV=End-diastolic volume, EF=Ejection fraction, LGE=Late-gadolinium enhancement, LV=Left ventricle, S'=Peak systolic strain rate.

## Conclusions

Early abnormal subclinical changes in vascular and myocardial function exist in newly diagnosed, treatment-naive RA patients, without overt changes in ventricular performance and geometry. The natural history of these observations and implications in the wider management of RA warrant further investigation.

## Funding

The study is funded by an EME project grant (11/117/27).

JPG and SP receive a research grant from Philips Healthcare.

SP is funded by British Heart Foundation fellowship (FS/10/62/28409).

